# Risk factors for postpartum haemorrhage in women with histologically verified placenta accreta spectrum disorders: a retrospective single-centre cross-sectional study

**DOI:** 10.1186/s12884-023-06103-5

**Published:** 2023-11-11

**Authors:** Naghmeh Ghaem Maghami, Fabrice Helfenstein, Gwendolin Manegold-Brauer, Gabriela Amstad

**Affiliations:** 1 Department of Obstetrics and Gynaecology, University Hospital Basel, Spitalstrasse 21, Basel, CH-4031 Switzerland; 2 Department of Clinical Research, University of Basel and University Hospital Basel, Basel, Switzerland

**Keywords:** Placenta accreta spectrum disorders, Postpartum haemorrhage, Risk factors, Blood loss

## Abstract

**Background:**

Placenta accreta spectrum (PAS) disorders have been reported with an increasing frequency of up to 3%. The increase in the incidence can be explained by the rising rate of Caesarean section (CS), assisted reproductive technology (ART) and previous uterine surgeries. PAS disorders are usually associated with postpartum haemorrhage (PPH). In our study, we investigated the risk factors for increased blood loss in women with histologically verified PAS disorders independent of delivery mode.

**Methods:**

In a retrospective single-centre cross-sectional study, 2,223 pregnant women with histologically verified PAS disorders were included. Risk factors for PPH in women with PAS disorders were examined and compared between women with PPH (study group; n = 879) and women with normal blood loss (control group; n = 1150), independent of delivery mode. PAS disorders were diagnosed histologically from the following specimens: placenta, placental-bed specimens, uterine curettage, uterine resection and/or total/partial hysterectomy. Medical data were extracted from clinical records of pregnant women with PAS disorders delivering at the University Hospital Basel between 1986 and 2019. The placenta data of women with PAS disorders were obtained and identified through a search from the database of the Department of Pathology, University Hospital Basel.

**Results:**

Between 1986 and 2019, there were 64,472 deliveries at the University Hospital Basel. PAS disorders were histologically verified in 2,223 women (2,223/64,472), and the prevalence of PAS disorders was 3.45%. A total of 879 women with PAS disorders showed PPH, independent of delivery mode (43.3%). Due to missing data for 194 women, the final analysis was conducted with the remaining 2,029 women. Placenta praevia (O.R. = 6.087; 95% CI, 3.813 to 9.778), previous endometritis (O.R. = 3.011; 95% CI, 1.060 to 9.018), previous manual placenta removal (O.R. = 2.530; 95% CI, 1.700 to 3.796), ART (O.R. = 2.169; 95% CI, 1.593 to 2.960) and vaginal operative birth (O.R. = 1.715; 95% CI, 1.225–2.428) can be considered important risk factors, and previous CS (O.R. = 1.408; 95% CI, 1.016 to 1.950) can be considered a moderate potential risk factor of PPH in women with PAS disorders.

**Conclusions:**

Placenta praevia, previous endometritis, previous placenta removal, ART and vaginal operative birth can be considered important risk factors of PPH in women with PAS disorders.

**Study registration:**

The study was registered under http://www.ClinicalTrials.gov (NCT05542043) on 15 September 2022.

**Supplementary Information:**

The online version contains supplementary material available at 10.1186/s12884-023-06103-5.

## Background

Placenta accreta spectrum (PAS) disorders, formerly known as morbidly adherent placenta, refers to a range of pathologic adherence of the placenta, including placenta accreta, increta and percreta [1, 2]. Variations in the lateral extension divide PAS disorders into focal, partial and total categories, depending on the number of placental cotyledons involved [1, 3]. PAS disorders also cover a wide spectrum of placental invasion with varied clinical significance. Histopathologic examination is considered the confirmatory gold standard, but it is not always carried out [4].

PAS disorders have become more common in recent years due to increased maternal age at delivery and increasing rates of uterine surgery [1, 3, 5–7]. It has also been described as the 20th -century iatrogenic disease because of the primary deciduo-myometrial defect in a scarred uterus as a result of a previous Caesarean section (CS), myomectomy, uterine curettage, hysteroscopic surgery or assisted reproductive technology (ART) [2, 3, 5–7].

PAS disorders are associated with increased maternal mortality and morbidity but do not contribute to adverse neonatal outcomes [8]. The main risk associated with any form of PAS disorder is massive obstetric haemorrhage, which leads to secondary complications, including coagulopathy, multisystem organ failure and death [9–16]. There are many known risk factors for PPH in women without PAS disorders, such as placenta praevia, previous uterine surgery, previous CS, hypertensive disorders, macrosomia, previous PPH, induction of labour, operative vaginal delivery, ART, multiple pregnancies, severe anaemia, uterine fibroma, placental abruption, obesity, prolonged third stage of delivery, non-use of oxytocin in the third stage, polyhydramnios and PAS disorders by themselves [13–15, 17–19]. Interestingly, some factors that have been traditionally considered risk factors for PPH, such as multiparity and increasing maternal age, have not been associated with PPH [12, 20–22]. There have been studies that attempted to create a prediction model or calculator that would estimate the risk for PPH individually [13]. However, further research is required to validate existing tools and develop a model for use in the general obstetric population. On the other hand, 22-61% of women who develop PPH have no risk factors, making it extremely difficult to predict which women will in fact develop PPH [12, 18, 23].

To our knowledge, there is no study about risk factors for PPH independent of delivery mode in women with PAS disorders. In the present study, we examined risk factors for PPH independent of delivery mode in pregnant women with histologically verified PAS disorders.

## Method

A retrospective single-centre cross-sectional study was conducted at the Department of Obstetrics and Antenatal Care, University Hospital Basel, Switzerland. A total of 2,223 pregnant women with histologically verified PAS disorders delivering at the University Hospital Basel between 1986 and 2019 were included in this study.

The study was conducted according to the guidelines of the Declaration of Helsinki and was approved by the Ethics Committee for Northwest and Central Switzerland in Basel (EKNZ ID 2020 − 01047). It was registered under http://www.ClinicalTrials.gov on 15 September 2022 (NCT05542043). If the person concerned provided a written or documented verbal refusal or was a pregnant woman under the age of 18, their health-related personal data or biological material were not used for this study.

The medical data were extracted from the patient’s clinical records. The placenta data of women with PAS disorders were obtained and identified through a search in the database of the Department of Pathology, University Hospital Basel.

The primary outcome was PPH (yes/no), as defined by the amount of blood loss during delivery. PPH was defined as blood loss of ≥ 500 ml following vaginal delivery or ≥ 1000 ml following CS [13, 16, 24]. During vaginal delivery, blood loss was measured using a blood loss bag, which we have been using for the last 6 years. Previously, blood loss was assessed subjectively, mostly by the amount of blood in the pads.

During a CS, blood loss has been measured by the amount of blood in the suction bag and in the pads. Pregnant women with histologically verified PAS disorders were divided into two groups: women with PPH (study group; *n* = 879) and women with normal blood loss (control group; *n* = 1,150), independent of delivery mode. Regardless of the delivery mode, placentas were sent for histologic examination in the following cases: antenatal bleeding, manual placenta removal or uterine curettage, PPH, chorioamnionitis, intrauterine growth restriction, poor neonatal outcome, multiples, preeclampsia/eclampsia, preterm birth, placenta praevia, neonatal death, abortion beyond the 14th week of pregnancy and stillbirth.

PAS disorders were diagnosed histologically from the following specimens: placenta, placental-bed specimens, uterine curettage, uterine resection and/or total/partial hysterectomy. For pathologic reporting, PAS disorders have been defined as abnormal implantation of chorionic villi on the superficial myometrium without an intervening decidual layer [25]. To categorize the extent of the placental bed’s involvement, we used the method Wortman [26] used. In that system, focal placenta accreta involves only one lobule, either partially or entirely; total placenta accreta involves all placental lobules; and partial placenta accreta involves at least two but not all placental lobules [26].

The following patient characteristics were recorded: maternal age, gravidity, parity, multiple pregnancy, body mass index (BMI), blood loss, gestational age, preterm birth, stillbirth, neonatal birth, delivery mode, tobacco use, placenta praevia, hypertension/preeclampsia, gestational diabetes (GDM), infection, bleeding during pregnancy, placental abruption, previous abortion curettage (before 12 weeks, between 12 and 18 weeks and after 18 weeks of gestation), previous hysteroscopic surgery or uterine curettage, previous CS, previous manual placenta removal, myomectomy, Asherman syndrome, ART, previous endometritis, ultrasound markers for PAS disorders, manual placenta removal or curettage during delivery.

Our primary objective was to examine whether and how the following potential risk factors were associated with PPH (the definitions used for each objective are provided in the cited references): increased maternal age at delivery (≥ 35 years); high gravidity (≥ 5); high parity (≥ 4); obesity (BMI ≥ 30 kg/m^2^); placenta praevia; previous uterine surgery (uterine curettage, abortion curettage, CS, hysteroscopic surgery, myomectomy, placental removal); previous abortion; Asherman syndrome; delivery mode; hypertensive disorders and preeclampsia [27]; tobacco use; placental abruption, defined as a premature separation of the placenta before delivery; ART; previous endometritis; multiples; GDM [28–31]; and infection in the current pregnancy (urinary tract infection, vaginal infection, chorioamnionitis, gastroenteritis, bronchitis and so on) [11, 13, 15, 18, 32–37]. Endometritis was defined as clinical symptoms in the birth report (fever, painful uterus and smelly lochia), increased inflammatory parameters in the blood and, in some cases, positive vaginal microbiological findings. Chorioamnionitis was defined by clinical symptoms (fever, maternal and/or foetal tachycardia), premature rupture of the membrane, increased inflammatory parameters in the blood and, in some cases, positive vaginal microbiological findings.

### Statistical analysis

#### Analysis set

A statistical analysis plan was conducted prior to the analysis. A sanity and validation test of the data was performed by a statistician. The analysis set was the available case set with patients having complete data concerning all potential risk factors. No imputation was done. The primary endpoint was postpartum haemorrhage (yes/no).

#### Statistical model and variable selection

For the primary analysis, a multivariable generalized linear model with binomial distribution of the error and a logit link function (logistic regression) was fitted to the data using PPH (yes/no) as the response variable and all the potential risk factors listed above as the explanatory variables. Starting with a full model including all the risk factors, a stepwise, bidirectional selection procedure based on the Akaike information criterion (AIC) was applied to select the potential risk factors that explained most of the probability of the occurrence of PPH. The AIC of the most parsimonious reduced model was then compared to the AIC of the full model and to the AIC of the null model (no risk factor, only describes the expected overall probability of occurrence of PPH). The best reduced model has the smallest AIC and hence the largest difference in AIC compared to the null and the full models. Only the odds ratios (O.R.) associated with the risk factors selected in the best reduced model are interpreted.

#### Primary analysis

The following potential risk factors were investigated for possible association with the probability of occurrence of postpartum haemorrhage: maternal age (3-level categorical: <35 years, 35–39 years, ≥ 40 years); gravidity (3-level categorical derived from a 6-level score: nulli-/primigravida, 2–4 gravida, ≥ 5 gravida); parity (3-level categorical derived from a 5-level score: nulli-/primipara, 2–3 para, ≥ 4 para); BMI (3-level categorical derived from a continuous variable: <30 kg/m^2^, 30 kg/m^2^-39.9 kg/m^2^, ≥ 40 kg/m^2^); placenta praevia (binary: yes/no); tobacco use (binary: yes/no); multiples (binary: yes/no); hypertension/preeclampsia (binary: yes/no); GDM (binary: yes/no); infection (binary: yes/no); abortion curettage < 12 weeks, 12–18 weeks, > 18 weeks (binary: yes/no); previous hysteroscopic surgery or uterine curettage (binary: yes/no); previous CS (binary: yes/no); previous manual placenta removal (binary: yes/no); myomectomy (binary: yes/no); Asherman syndrome (binary: yes/no); ART (binary: yes/no); previous endometritis (binary: yes/no); and mode of delivery (4-level categorical: nonoperative vaginal delivery, operative vaginal delivery, elective CS and emergent CS).

#### Statement about P value

*P* values are not provided for several reasons. First, *P* values are too often used as a dichotomy to decide whether a factor is statistically significant. This practice is incorrect and leads to spurious conclusions [38]. Second, model selection, as planned here, does not allow for a meaningful calculation and interpretation of *P* values associated with the factors’ estimates. Consequently, to assess the strength of the association between a given potential risk factor and the occurrence of PPH, we used the following criteria:

1. The size of the estimates; here, the O.R. An O.R. around 1 indicates a weak or no association between the risk factor and the probability of occurrence of PPH. The further the O.R. departs from 1, the stronger the association.
2. The size of the 95% confidence interval (CI) associated with the estimate. The narrower the 95% CI, the stronger the conclusion about the association. A large O.R. with a large range in the 95% CI may be regarded as inconclusive regarding the existence of a true association.

## Results

Between 1986 and 2019, there were 64,472 deliveries at the University Hospital Basel. PAS disorders were histologically verified in 2,223 women (2,223/64,472). The prevalence of PAS disorders was 3.45%; specifically, the prevalence was 1.4% (712/51,278) between 1986 and 2014 and 11.5% (1,511/13,194) between 2014 and 2019. Due to missing data for 194 pregnant women (1 woman under 18 years of age, 189 women with missing data on BMI, 1 woman with missing data on tobacco use, 1 woman with missing data on abortion curettage after 18 weeks of pregnancy and 1 woman with missing data on ART), the final analysis included 2,029 women.

Table 1 shows the demographic characteristics, the delivery mode, the pregnancy characteristics and the blood loss of the women in our sample, stratified by group (control vs. PPH). In our sample of women with PAS disorders, 879 women had PPH (43.3%) and 1150 had normal blood loss (56.7%). In the study group, there were more vaginal deliveries (53.0% vs. 17.7% in the control group), especially operative vaginal delivery (213 (24.2%) vs. 57 (5.0%); O.R. = 1.715; 95% CI, 1.223 to 2.429). There was a significant difference in the rate of CS between groups (22.8% vs. 77.3%); in particular, non-elective CS was significantly higher in the control group (86 (9.8%) vs. 472 (41.0%); O.R. = 0.064; 95% CI, 0.047 to 0.086). The mean gestational age at delivery was 39/2 ± 2.5 weeks in women with PPH and 38/3 ± 2.5 in women without PPH.

**Table 1:** Demographic and pregnancy characteristic

			Women with PPH		Women without PPH
			(n = 879)		(n = 1150)
Maternal age (SD) (years)			33.1 (5.0)		33.5 (5.4)
Age (%)		< 35 years	538 (61.2)		658 (57.2)
		35–39 years	264 (30.0)		339 (29.5)
		≥ 40 years	77 (8.8)		153 (13.3)
Gravidity (IQR)			2 (1–3)		2 (1–3)
Gravidity (%)		0–1 gravida	347 (39.5)		483 (42.0)
		2–4 gravida	488 (55.5)		600 (52.2)
		≥ 5 gravida	44 (5.0)		67 (5.8)
Parity (IQR)			1.0 (1–2)		1.0 (1–2)
Parity (%)		0–1 para	489 (55.6)		716 (62.3)
		2–3 para	368 (41.9)		403 (35.0)
		≥ 4 para	22 (2.5)		31 (2.7)
BMI (SD) (kg/m^2^)			28.1 ± 4.7		28.9 ± 5.3
BMI (%)		< 30 kg/m^2^	615 (70.0)		726 (63.1)
		30-39.9 kg/m^2^	241 (27.4)		382 (33.2)
		≥ 40 kg/m^2^	23 (2.6)		42 (3.7)
Smoking (%)			82 (9.3)		100 (8.7)
Singleton pregnancy (%)			773 (87.9)		931 (81.0)
Multiples (%)			106 (12.1)		219 (19.0)
Placental abruption (%)			8 (1.7)		31 (3.7)
Placenta praevia (%)			60 (6.8)		44 (3.8)
Hypertension/Preeclampsia (%)			73 (8.3)		158 (13.7)
GDM (%)			97 (11.0)		140 (12.2)
Infection in pregnancy (%)			116 (13.2)		203 (17.7)
Bleeding in pregnancy (%)			142 (16.2)		143 (12.4)
Blood loss (IQR) (ml)			1200 (900–1700)		500 (400–600)
Previous abortion curettage (%)			252 (28.7)		372 (32.3)
Abortion curettage < 12 weeks (%)			267 (30.4)		392 (34.0)
Abortion curettage 12–18 weeks (%)			34 (3.9)		45 (3.9)
Abortion curettage > 18 weeks (%)			13 (1.5)		23 (2.0)
Previous hysteroscopic surgery/ curettage (%)			56 (6.4)		65 (5.7)
Previous Caesarean section (%)			106 (12.1)		205 (17.8)
Previous myomectomy (%)			13 (1.5)		24 (2.1)
Asherman syndrome (%)			8 (0.9)		16 (1.4)
Previous manual placenta removal (%)			103 (11.7)		69 (6.0)
Assisted reproductive technology (%)			142 (16.2)		202 (17.6)
Previous endometritis (%)			14 (1.6)		8 (0.7)
Suspected ultrasound markers (%)			36 (4.1)		19 (1.7)
Preterm birth (%)			176 (20.0)		409 (35.6)
Neonatal death (%)			1 (0.2)		1 (0.1)
Vaginal non-operative delivery			466 (53.0)		203 (17.7)
Vaginal operative delivery			213 (24.2)		57 (5.0)
Elective Caesarean section			114 (13.0)		418 (36.3)
Non-elective Caesarean section			86 (9.8)		472 (41.0)

Categorical variables are presented as counts and (percentages)

Continuous variables are expressed as mean and (standard deviation)

Non-normally distributed variables are presented with their median and (Inter-Quartile Range)

### Primary analysis

Table 2 compares the AICs of the null, full and best reduced models.

**Table 2:** Comparison of the fit of the null, full and best models (after stepwise selection based on AIC) to explain variation in the probability of occurrence of PPH

Model	Deviance	Model df	Residual df	AIC	Delta AIC	n
Best model	2011.77	11.00	2017	2035.77	0.00	2029
Full model	1999.72	27.00	2001	2055.72	19.95	2029
Null model	2776.49	0.00	2028	2778.49	742.72	2029

The differences in AIC between the best reduced model and the full and null models are large and indicate that the best model better explains the variation in the probability of occurrence of PPH than the null and full models. Table 3 shows the risk factors that were retained in the best reduced model after stepwise bidirectional selection.

**Table 3:** Best model to explain variation in the probability of occurrence of PPH.

	Odds ratio	CI
Placenta praevia	6.087	[3.813–9.778]
Previous endometritis	3.011	[1.060–9.018]
Previous manual placenta removal	2.530	[1.700-3.796]
Assisted reproductive technology	2.169	[1.593–2.960]
Previous Caesarean section	1.408	[1.016–1.950]
Infection in pregnancy	0.696	[0.511–0.946]
Asherman syndrome	0.405	[0.138–1.113]
Previous abortion curettage > 18 weeks	0.268	[0.114–0.612]
Vaginal operative delivery	1.715	[1.225–2.428]
Elective Caesarean section	0.064	[0.046–0.088]
Non-elective Caesarean section	0.064	[0.047–0.086]

All factors in the best model help explain the overall variation in the occurrence of PPH although some factors contribute only very modestly whereas others are stronger risk factors. Some odds ratios have a large value and a large range in the 95% CI, making it difficult to conclude that there is an association between the risk factor and the occurrence of PPH.

Based on the estimated O.R. and associated 95% CI, the following factors can be considered risk factors with strong evidence of a moderate (O.R. > 1.5) to strong association (O.R. > 2) with the probability of occurrence of PPH: placenta praevia (O.R. = 6.087; 95% CI, 0.778 to 3.813), previous endometritis (O.R. = 3.011; 95% CI, 1.060 to 9.018), manual placenta removal in the previous pregnancy (O.R. = 2.530; 95% CI, 1.700 to 3.796), ART (O.R. = 2.169; 95% CI, 1.593 to 2.960) and vaginal operative delivery (O.R. = 1.715; 95% CI, 1.225 to 2.428). There were more women with concomitant placenta praevia and previous CS in the group with PPH (24//879 vs. 9/1150) (O.R. = 3.551; 95% CI, 1.244 to 1.899).

Based on the O.R. and the range of the associated 95% CI, CS in the previous pregnancy (O.R. = 1.408; 95% CI, 1.016 to 1.950) can be considered a potentially moderate risk factor. However, due to uncertainty in the point estimates, the strength of the association with the probability of occurrence of PPH remains unknown in both cases and needs to be confirmed with further investigations.

The following factors are associated with a decrease in the probability of occurrence of PPH: infection in pregnancy (O.R. = 0.696; 95% CI, 0.511 to 0.946) (a large uncertainty remains regarding the true strength of the association) and CS in the current pregnancy (O.R. = 0.064; 95% CI, 0.046 to 0.088).

### Sensitivity analysis

The best model using age and BMI as continuous variables completely aligns with the best model using age and BMI as categorical variables. The supplementary material includes the risk factors that were retained in the best reduced model using age and BMI as continuous variables after stepwise bidirectional selection.

## Discussion

The prevalence of PAS disorders in our study was 3.45% and aligns with the prevalence reported in the literature [1, 6, 7]. However, the prevalence has dramatically increased, from 1.4 to 11.5%, at our hospital in recent years. This is approximately a tenfold increase and is in accordance with the increase in CS rates from less than 10% to over 30% [1, 6, 7, 39]. On the other hand, the prevalence of PAS disorders in the University Hospital Basel does not reflect the incidence in the general population.

Our study was an examination of the risk factors for PPH independent of delivery mode in women with histologically verified PAS disorders. Wright et al. investigated the predictors of massive blood loss (≥ 5000 ml) in women with PAS disorders who had undergone a hysterectomy [19]. There was no association between massive blood loss and maternal age, gravidity, number of previous deliveries, number of previous CSs or degree of placental invasion [19]. In a large multicentre US cohort study [5], the risk of PAS disorders was 7 times greater after one prior CS, 56 times greater after three or more CSs and 6 times greater after prior PPH. In our study, placenta praevia, previous placenta removal, non-surgical factors such as ART and previous endometritis were identified as risk factors with strong evidence of moderate to strong association with PPH in women with histologically verified PAS disorders.

Placenta praevia is the most important risk factor for PAS disorders and PPH [3, 5, 15, 36]. Thurn et al. [5] found that placenta praevia was the single most important risk factor for PAS disorders and was reported in 49% of all cases of PAS disorders. Thurn et al. [5] concluded that in women presenting with placenta praevia and previous CS, the risk of PAS disorders ranged from 3% for one previous CS to 61% for five CSs. This suggests tropism of the blastocyst in the uterine scar tissue, and women with a prior CS presenting with a low-lying placenta or placenta praevia represent the group with the highest risk for PAS disorders [3]. This epidemiologic association also indicates that a previous uterine scar can affect implantation and placentation [3]. In a systematic review and meta-analysis from 2020, placenta praevia was present in 92.8% of pregnancies complicated by posterior PAS disorders [40]. On the other hand, placenta praevia is also an important risk factor for PPH because the lower uterine segment is only weakly contractile; therefore, the primary mechanism of preventing blood loss from the placental implantation site is ineffective [3, 7, 12, 15, 17]. In cases of PAS disorders, the placenta actually invades the myometrium. There is no plane of cleavage at which the placenta can be separated, which leads to heavy haemorrhage [15].

In the present study, we found more PPH in women with PAS disorders who gave birth vaginally, particularly through vaginal operative delivery. First, this reflects the varying severity of placental disease and hence the related varying antenatal recognition. We suspect there are two clinically diverse groups of women with PAS disorders. The first group consists of women with significant amounts of placental tissue that invades the uterine wall. These women are recognised antenatally and get scheduled delivery with many benefits and minor blood loss. The second group consists of women with focal placenta accreta, which is mostly not recognised antenatally. These women often give birth vaginally, are unrecognised until the time of delivery and show more PPH. On the other hand, vaginal operative delivery per se is a risk factor for PPH in women without PAS disorders, and manual placenta removal per se is also an intervention associated with increased blood loss [12, 20–22]. A second possible explanation is that removal of the placenta during a CS is easier than during vaginal delivery.

ART was first linked to PAS disorders in 2011 [41], and this association has been confirmed in a meta-analysis [42]. It is generally assumed that PAS disorders with ART result from maternal factors such as maternal age and uterine factor infertility rather than the procedures [43]. Kaser et al. [44] found that there is an independent relationship between PAS disorders and ART even after they controlled for maternal factors and placenta praevia. Nyfløt et al. [14] showed that ART is a risk factor for severe PPH. In our study, ART can be considered a risk factor for PPH independent of delivery mode in women with PAS disorders. However, further studies are needed to examine this association, and future studies of this mechanism may provide an opportunity to reduce PAS risk during the ART process.

Previous endometritis was associated with 3.011 times greater probability and previous manual placenta removal with 2.530 times greater probability of PPH in the study group. Our results are consistent with those of previous studies [5, 45]. However, it is to be noted that only previous manual placenta removal was investigated in our study, not explicitly previous PPH.

We cannot logically explain the decrease in blood loss in Asherman syndrome. Asherman syndrome occurs when trauma to or removal of the basal layer of the endometrium occurs in opposing areas in the uterine cavity. Histologically, Asherman syndrome is a fibrosis of the endometrium, and one of the typical symptoms is hypomenorrhea. The possible cause of decreased bleeding in pregnancy may be a smaller “active surface of the uterus” and thus the smaller adhesion surface for the placenta. Although, on the other hand, there is a little information about changes in the uterine myometrium in Asherman syndrome. Hypothetically, chronic inflammation of the uterine myometrium with excessive accumulation of extracellular matrix components produced by fibroblasts could lead to the development of diffuse fibrosis, in which connective tissue replaces normal parenchymal tissue and impairs uterine vascularization. This could secondarily cause a reduction of blood loss in PAS. Unlike chronic inflammation, acute endometritis is treated with antibiotics and is characterized by an accumulation of pathogens and white blood cells in the connective tissue by increasing blood circulation and thus improving the removal of damaged cells. Therefore, acute endometritis does not progress normally to chronic inflammation with fibrosis formation. Although previous endometritis is known as a risk factor for PAS, little is known about its effect on bleeding. Based on the O.R. in our study, previous endometritis can be considered a potentially strong risk factor for PPH. However, further prospective studies are needed to investigate the relationship between PPH and acute/chronic uterine inflammation in women with PAS.

Our study has some weaknesses. First, it employs a retrospective design, which is also why many important data are missing or were not investigated. Examples are the lack of information on prenatal suspicion of PAS by ultrasound; the lack of objective measurement of blood loss because objective measurement of blood loss using a blood loss bag has been used for the last few years; the lack of investigation of known risk factors for PPH, such as macrosomia, induction of labour, severe anaemia, uterine fibroma, prolonged third stage of delivery, non-use of oxytocin in the third stage of delivery, recurrence of uterine surgery and polyhydramnios; and the lack of exact management of PPH in older data. The steadily improving prenatal diagnosis of PAS, the routine implementation of the DACH guidelines and the involvement of a multidisciplinary team improve the management of PPH with reduced blood loss and maternal morbidity and mortality in the last years. The second disadvantage is the long period over which the study was conducted. Risk factors for PPH, such as a CS, placenta praevia, curettage and manual removal of the placenta, were less common 15–20 years ago than in the last 10 years. On the other hand, not all placentas from women who gave birth in this period were examined, so a certain portion of PAS disorders has been undiagnosed. Because of these weaknesses - retrospective data of a single-centre over 30 years - this must be considered when we present these data, and the results must be used with care.

In conclusion, this study provides strong evidence that placenta praevia, previous manual placenta removal, ART, previous endometritis and vaginal operative delivery can be considered risk factors for PPH in women with PAS disorders. However, further studies are needed to investigate the impact of risk factors for PPH in PAS disorders in a time-dependent manner, the impact of repeated uterine surgery, the use of ART, the role of ultrasound in the prenatal detection of PAS and the impact of other variables on blood loss in women with PAS disorders. Our results could initiate the planning and conducting of prospective powered studies to verify the potential risk factors for PPH in this high-risk group of women.

## Conclusions

Placenta praevia, previous endometritis, previous placenta removal, assisted reproductive technology and vaginal operative birth can be considered important risk factors of PPH in women with PAS disorders.

### Electronic supplementary material

Below is the link to the electronic supplementary material.

Supplementary Material 1

## Data Availability

The datasets used and analysed during the current study are available from the corresponding author on reasonable request.

## List of abbreviations

AIC: Akaike information criterion
ART: Assisted reproductive technology
BMI: Body mass index
CI: Confidence Interval
CS: Caesarean Section
GDM: Gestational diabetes
O.R: Odds Ratio
PAS: Placenta accreta spectrum
PPH: Postpartum haemorrhage
