# Supplementary material for: Risk factors for postpartum haemorrhage in women with histologically verified placenta accreta spectrum disorders: a retrospective single-centre cross-sectional study
Source: BMC Pregnancy Childbirth. 2023 Nov 11;23:786. doi: 10.1186/s12884-023-06103-5 (PMC10638773; doi:10.1186/s12884-023-06103-5)
Supplement: Supplementary file 1 — Supplementary Material 1 [file 12884_2023_6103_MOESM1_ESM.docx]

**Sensitivity analysis**

Comparison of the fit of the null, the full and the best model (after stepwise selection based on AIC) using maternal age and BMI as continuous variables to explain variation in the probability of occurrence of PPH.

| Model | Deviance | Model df | Residual df | AIC | Delta AIC | n |
| --- | --- | --- | --- | --- | --- | --- |
| Best model | 2011.77 | 11.00 | 2017 | 2035.77 | 0.00 | 2029 |
| Full model | 2004.34 | 25.00 | 2003 | 2056.34 | 20.57 | 2029 |
| Null model | 2776.49 | 0.00 | 2028 | 2778.49 | 742.72 | 2029 |

**Best model to explain variation in the probability of occurrence of PPH when using age and BMI as continuous variables.**

|  | Odds ratio | CI |
| --- | --- | --- |
|  |  |  |
|  |  |  |
| Placenta praevia | 6.087 | [3.813-9.778] |
| Previous endometritis | 3.011 | [1.060-9.018] |
| Previous manual placenta removal | 2.530 | [1.700-3.796] |
| Assisted reproductive technology | 2.169 | [1.593-2.960] |
| Previous Caesarean section | 1.408 | [1.016-1.950] |
| Infection in pregnancy | 0.696 | [0.511-0.946] |
| Asherman syndrome | 0.405 | [0.138-1.113] |
| Previous abortion curettage > 18 weeks | 0.268 | [0.114-0.612] |
| Vaginal operative delivery | 1.715 | [1.225-2.428] |
| Elective Caesarean section | 0.064 | [0.046-0.088] |
| Non-elective Caesarean section | 0.064 | [0.047-0.086] |
|  |  |  |
